# Recent advances in mass spectrometry-based proteomics and metabolomics in chronic rhinosinusitis with nasal polyps

**DOI:** 10.3389/fimmu.2023.1267194

**Published:** 2023-09-06

**Authors:** Shudi Guo, Ming Tian, Yunping Fan, Xiangyang Zhang

**Affiliations:** ^1^ School of Pharmaceutical Science and Technology, Tianjin University, Tianjin, China; ^2^ Department of Otolaryngology, The 7th Affiliated Hospital of Sun Yat-sen University, Shenzhen, China

**Keywords:** chronic rhinosinusitis, proteomics, metabolomics, mass spectrometry, biomarker

## Abstract

Chronic rhinosinusitis with nasal polyps (CRSwNP) is a complex and heterogeneous disease, typically diagnosed through endoscopy and computed tomography and treated with glucocorticoid or surgery. There is an urgent need to develop molecular-level diagnostic or prognostic tools to better understand the pathophysiology of CRSwNP. Proteomics and metabolomics, emerging fields, offer significant potential in elucidating the mechanisms underlying CRSwNP. Mass spectrometry, a powerful and sensitive tool for trace substance detection, is broadly applied for proteomics and metabolomics analysis in CRSwNP research. While previous literature has summarized the advancement of mass spectrometry-based CRSwNP proteomics from 2004 to 2018, recent years have seen new advances in this field, particularly about non-invasive samples and exosomes. Furthermore, mass spectrometry-based CRSwNP metabolomics research has opened new avenues for inquiry. Therefore, we present a comprehensive review of mass spectrometry-based proteomics and metabolomics studies on CRSwNP conducted between 2019 and 2022. Specifically, we highlight protein and metabolic biomarkers that have been utilized as diagnostic or prognostic markers for CRSwNP. Lastly, we conclude with potential directions for future mass spectrometry-based omics studies of CRSwNP.

## 1 Introduction

Chronic rhinosinusitis (CRS) is a prevalent disease characterized by nasal blockage, nasal discharge, facial pain, and loss of smell, lasting 12 weeks or longer (1). It affects 5-12% of the general population and can be triggered by various factors, including genes, family history, region, air pollution, occupational exposures, and smoking tobacco (2). Moreover, CRS is associated with asthma (3), allergy (4), and other diseases, leading to an increased health burden on patients.

Based on the presence or absence of nasal polyps, CRS is divided into chronic rhinosinusitis with nasal polyps (CRSwNP) and chronic rhinosinusitis without nasal polyps (CRSsNP). CRSwNP is characterized by a T-helper (Th) 2 predominant inflammatory pattern, while CRSsNP is characterized by a Th1 predominant inflammatory pattern (5). Furthermore, CRSwNP can be categorized as eosinophilic CRSwNP (eCRSwNP) or non-eosinophilic CRSwNP (neCRSwNP) based on the percentage of tissue eosinophils, with eCRSwNP exhibiting a higher recurrence rate than neCRSwNP (6, 7). However, these classifications fail to fully reflect the cellular or molecular immunological profile. Hence, it is necessary to diagnose and classify CRS subtypes at the molecular level. One study has indicated geographical and ethnic differences in CRSwNP (8). Moreover, the diagnostic criteria for eCRSwNP, that is, the cutoff value for the percentage of eosinophils per high-pressure field in tissue samples, ranged from 5 to 50. However, the European Position Paper on Rhinosinusitis and Nasal Polyps 2020 (EPOS 2020) suggested that the cutoff value should be 10 or higher.

Proteomics and metabolomics have emerged as promising fields for diagnosing diseases, including CRS (9–11). Compared with genes and RNA, proteins are the ultimate carriers of biological functions, and the proteome represents the state of organisms performing direct functions, reflecting the true expression of gene transcription; whereas the metabolome is the downstream of the transcription-expression network as well as the protein-action network, which can reflect changes in the state of the phenotypes of the organisms and explore the metabolic mechanisms of the whole organism. Therefore, proteomics and metabolomics can help to better understand the pathophysiology of CRSwNP. For the discovery of disease biomarkers, proteins and metabolites are intuitive and easily detectable molecules. The proteomic samples of CRSwNP include nasal polyps, nasal mucosa, and nasal mucus (e.g., nasal swabs, and nasal lavage fluid), while metabolomic samples are mainly polyp tissues and nasal mucosa. Both proteomic and metabolomic analysis of CRSwNP have made significant progress. Particularly, in proteomics, a study systematically summarized proteomic studies on nasal mucus and nasal mucosa from 2004 to 2018 and identified 2962 validated proteins (12), some of which are already utilized as diagnostic markers (13). Notably, the shift from invasively obtained (e.g., nasal polyps) to non-invasively obtained (nasal secretions) proteomic samples have reduced the risk of patient infections, while also reflecting the disease status (14).

Furthermore, mass spectrometry techniques (MS) (14) have been widely used in CRS proteomics and metabolomics, providing high resolution, high throughput, and mass accuracy for the clinical disease analysis. Many studies have reported on the diagnostic, therapeutic, or prognostic role of mass spectrometry in various diseases (10, 15). The most commonly used mass spectrometry platform is liquid chromatography combined with tandem mass spectrometry (LC-MS/MS). For proteomics specifically, data acquisition modes include data-dependent acquisition (DDA) and data-independent acquisition (DIA). DDA, a traditional technology, has been extensively used in proteomics. The traditional DIA proteomic method involves using DDA for building a spectrum library and DIA is used to obtain protein profiles. Recently, a novel proteomic method incorporated parallel reaction monitoring (PRM) scanning after traditional DIA to verify key proteins. This method has been successfully applied in CRSwNP (16).

In recent years, significant progress has been made in proteomics and metabolomics of CRSwNP, with numerous proteins and metabolites being used as biomarkers. In this review, we provide a summary of mass spectrometry-based proteomic and metabolomic studies in CRSwNP from 2019 to 2022, spotlighting proteins and metabolites that are used as biomarkers. Additionally, we provide future directions for CRSwNP research.

## 2 Mass spectrometry-based proteomics of chronic rhinosinusitis with nasal polyps

### 2.1 Sample collection

Nasal samples include tissues (i.e., nasal polyps and mucosa) and mucus (e.g., nasal secretions, and nasal lavage fluid). Several protein extraction methods are available for diverse samples, and here we just introduce the main workflow of protein extraction.

#### 2.1.1 Tissue

Tissue samples include nasal polyps and nasal mucosa (17, 18). Generally, tissues obtained surgically need to be immediately frozen with liquid nitrogen or dry ice until extracting protein. The main steps of extracting proteins from tissues are as follows (19), initially, an equivalent weight of tissue is weighed, lysis solution is added, and homogenization is performed using a tissue grinder. Subsequently, centrifugation is performed to obtain the supernatant. Finally, the protein concentration is measured.

#### 2.1.2 Mucus

As a non-invasively obtained sample, mucus is often used as a subject for proteomic analysis of CRSwNP. Mucus can be obtained in several ways, Parra-Ferro et al. summarized the methods used for nasal mucus collection in recent years and discussed the advantages and shortcomings of each method (20). Polyurethane foam, commonly used for mucus collection, has a high secretion and protein recovery (21). Filter paper, used for collecting nasal secretions, is highly specific for olfactory binding proteins (14). For nasal lavage fluid, normal saline is instilled into the middle meatus of the nasal cavity using a syringe, then, lavage fluid is recollected into a sterilized conical tube (22). Following collection, the main protein extraction steps from mucus are as follows, ultrapure water is added to the mucus extraction vessel (except for nasal lavage fluid), mixed at room temperature, centrifuged, and the supernatant collected for protein concentration measurement.

### 2.2 Applications of mass spectrometry-based proteomics in chronic rhinosinusitis with nasal polyps

Proteomics is a powerful tool to reveal changes in proteins at the molecular level in CRSwNP patients. Numerous articles have reported on proteomic analysis of CRSwNP, which have identified differentially expressed proteins (DEP) that are associated with immune responses, programmed cell death, and vesicle-mediated transport (19, 23). Recently, new progress has been made in the proteomics of CRS. Here, we summarize mass spectrometry-based proteomics studies of CRSwNP from 2019 to 2022 and highlight proteins as biomarkers for diagnosis or prognosis (**Table 1**).

**Table 1:** Mass spectrometry-based proteomics studies of CRSwNP from 2019 to 2022.

Sample	MS method	Study findings	Reference
Nasal secretions CRSwNP vs CRSsNP vs Control	LC-ESI-Q-Orbitrap-MS	The expression of interleukin (IL)-7, IL-9, IL-17A, and IL-22 and neutrophil-mediated immune responses are significantly increased in CRSwNP.	Kim et al. (14)
Nasal secretion CRSwNP vs CRSsNP	LC-ESI-Q-Orbitrap-MS	Antibiotics could have stronger effects on their associations in patients with CRSwNP than in CRSsNP	Kim et al. (24)
Nasal lavage fluid CRSwNP vs CRSsNP	LC-ESI-Q-TOF-MS	Mucin 5AC is upregulated in CRSwNP patients, and it may promote tissue remodeling and angiogenesis in nasal polyps, therefore, it may be a potential therapeutic target for nasal polyps	Wang et al. (25)
Nasal lavage fluid, tissue, cells CRS vs Control	LC-MS	Apolipoprotein E and periostin are potential biomarkers of nasal mucosal inflammation	Chung et al. (22)
Nasal lavage fluid, cells CRSwNP vs Control	LC-MS	Exosomes may affect cellular proliferation or cell remodeling in the progression of CRSwNP through p53 signaling pathways	Zhou et al. (26)
Nasal mucus CRSwNP vs CRSsNP vs Control	LC-ESI-Q-TOF-MS	The tissue remodeling pathway and the epithelial-to-mesenchymal transition biologic process are more significantly upregulated in CRSwNP patients compared to CRSsNP and control.	Kao et al. (27)
Olfactory cleft mucus CRSwNP vs CRSsNP vs Control	Nano-LC-ESI-Orbitrap-MS	Lipocalin-1 and Lipocalin-15 are thought to play a role in odorant binding, and glutathione S-transferases, alcohol dehydrogenases, and other oxidizing enzymes, are thought to play a role in odorant metabolism are downregulated in CRSwNP.	Soler et al. (28)

LC-ESI-Q-Orbitrap-MS, liquid chromatography/electrospray ionization quadrupole orbitrap mass spectrometry; LC-ESI-Q-TOF-MS, liquid chromatography/electrospray ionization quadrupole time-of-flight mass spectrometry; LC-MS, liquid chromatography/mass spectrometry; Nano-LC-ESI-Orbitrap-MS, nano liquid chromatography/electrospray ionization orbitrap mass spectrometry.

Kim’s team has been working on the proteomics of CRSwNP. They performed DDA and DIA proteomic analysis on nasal secretions from CRSwNP, CRSsNP, and normal control, respectively, and identified 2020 proteins and the expression of interleukin (IL)-7, IL-9, IL-17A and IL-22 and neutrophil-mediated immune responses were significantly increased in CRSwNP compared to control (14). This result is consistent with the regional specificity of CRSwNP and provides new insights into the endotypes of CRS. Additionally, they studied the relationship between the nasal microbiome and nasal secretion proteome in CRSwNP patients after taking antibiotics 3 months ago. They found that antibiotics could have stronger effects on their associations in patients with CRSwNP than in CRSsNP (24).

Additionally, some potential diagnostic biomarkers have been found in recent studies, and different studies have obtained similar findings confirming the confidence. Wang et al. collected exosomes in nasal lavage fluid and found that mucin 5AC is upregulated in CRSwNP patients. Then, they discovered that mucin 5AC enriched exosomes from CRSwNP patients could increase the expression of cyclooxygenase-2 (COX-2), vascular endothelial growth factor (VEGF), and matrix metalloproteinase-9 (MMP-9) in CRSsNP-derived fibroblasts. They reported that mucin 5AC may promote tissue remodeling and angiogenesis in nasal polyps and therefore, it may be a potential therapeutic target for nasal polyps (25). Additionally, Workman et al. studied the CRSwNP transcriptome and proteome by the SomaLogic SOMAscan platform and found that messenger RNA (mRNA) expression differences differed more from protein expression differences and that predicting mechanistic changes in disease from mRNA levels alone is less reliable (29). Several genes canonically thought to be overexpressed in CRSwNP, including IL-5, IL-13, thymic stromal lymphopoietin (TSLP), chemokine C-C motif ligand (CCL)13, and CCL26, showed substantial increases in mRNA transcription, but had minimally or unchanged protein expression. Others, including immunoglobulin (Ig)E, periostin, CCL18, and cystatin (CST)1/2, were increased at both the transcriptomic and proteomic levels. Chung et al. found that apolipoprotein E and periostin are potential biomarkers of nasal mucosal inflammation by *in vivo* and *in vitro* analysis (22). This study has important implications for the clinical diagnosis of nasal mucosal inflammatory diseases, such as CRS, and future attention should be paid to the practical application to clinical diagnosis. Similarly, a study by Ninomiya et al. found that periostin could be a novel biomarker for postoperative recurrence of CRSwNP (13). Both of these studies confirmed the usefulness of periostin in nasal inflammation. In addition, for recurrent CRSwNP, Wang et al. found elevated levels of eotaxin, IL-17A, and regulated upon activation normally T expressed and presumably secreted (RANTES) in recurrent patients compared to primary patients by extracting cytokines from their serum. Notably, serum eotaxin (Area under the curve (AUC) = 0.729) and RANTES (AUC = 0.776) showed a pronounced ability to predict CRSwNP postoperative recurrence (30). Therefore, eotaxin and RANTES might serve as potential prognostic biomarkers for postoperative recurrence.

To understand the pathological process of CRSwNP in-depth, Kao et al. found that patients with CRS have dysfunctional immune pathways, reduced cell signaling, increased cell metabolism, and associated tissue remodeling pathways by comparative proteomic analysis of mucus from normal subjects and patients with CRS (27). In particular, the tissue remodeling pathway and the epithelial-to-mesenchymal transition biologic process were more significantly upregulated in CRSwNP patients compared to CRSsNP. Loss of smell is a main symptom of CRS, and alterations of olfactory cleft (OC) mucus may reflect olfactory dysfunction in CRS patients. Soler et al. studied OC mucus proteins from CRSwNP, CRSsNP, and controls. They found that Lipocalin-1 and Lipocalin-15 which are thought to play a role in odorant binding, and glutathione S-transferases (GSTs), alcohol dehydrogenases, and other oxidizing enzymes, which are thought to play a role in odorant metabolism were downregulated in CRSwNP (28). Their findings suggest OC mucus dysregulation may indeed be associated with olfactory dysfunction in CRS patients.

Furthermore, more and more studies focused on exosomes. Muellere et al. isolated exosomes from nasal mucus, performing exosomes proteome analysis by SOMAscan™ platform, then they found that cystatin-SN, peroxiredoxin-5, and glycoprotein V showed good accuracy for predicting CRSwNP (AUC> 0.99). The result also has been confirmed in tissue samples (31). Zhou et al. reported that exosomes may lead to the remodeling of the sinonasal mucosa in patients with CRSwNP (26). And the key protein signaling pathway was p53.

In conclusion, recent proteomic studies of CRSwNP have revealed some potential diagnostic biomarkers and therapeutic biomarkers. In recent years, most study samples are non-invasively obtained mucus, which is more convenient and less invasive. Exomics, a new field of proteomics, has also achieved some progress in CRSwNP proteomics. At the parallel, we can see an increasing number of studies focusing on the relationship between the transcriptome, the nasal microbiome, and the proteome. Multi-omics analysis can reveal the complex physiological and pathological processes of CRSwNP and provide a better understanding of the disease.

## 3 Mass spectrometry-based metabolomics of chronic rhinosinusitis with nasal polyps

### 3.1 Sample collection

Most samples for CRSwNP metabolomics are tissues (i.e., nasal polyps, nasal mucosa). These samples could reflect the local pathophysiological changes in patients with CRSwNP. To avoid metabolite degradation, tissues obtained surgically need to be immediately frozen with liquid nitrogen or dry ice. The metabolite extraction protocols for tissue samples are similar across different labs. Chetwynd et al. described in detail the tissue sample preparation for metabolomics (32). One thing that should be noted is that the weight of the tissue samples used for extraction can only vary by 5-10%, which is important for parallel experiments and later mass spectrometry analysis. The extraction reagents used in recent experiments include 4 mL/g of cold methanol and 0.85 mL/g of cold water (33, 34), cold methanol (35, 36), and cold methanol/acetonitrile/H_2_O at 2:2:1 (v/v/v) (37).

### 3.2 Applications of mass spectrometry-based metabolomics in chronic rhinosinusitis with nasal polyps

Metabolomics in CRSwNP is an emerging field and is less extensive than proteomics. Currently, most metabolomics studies in CRSwNP utilize an untargeted approach to identify differentially expressed metabolites, aiming to reveal the underlying molecular-level pathological processes of the disease. Here, we summarize mass spectrometry-based metabolomics studies of CRSwNP from 2019 to 2022 and highlight metabolites as biomarkers for diagnosis or prognosis (**Table 2**).

**Table 2:** Mass spectrometry-based metabolomics studies of CRSwNP from 2019 to 2022.

Sample	MS method	Study findings	Reference
Plasma, nasal secretions CRSwNP vs Control	LC-MS/MS	Maresins could be diagnostic and therapeutic biomarkers.	Beegun et al. (38)
Tissue, blood eCRSwNP vs Control	LC-MS/MS	Enzymes and metabolites associated with fatty acid metabolism may be potential therapeutic targets.	Miyata et al. (39)
Tissue CRSwNP vs CRSsNP vs Control	LC-MS/MS	Dysregulated specialized pro-resolving lipid mediators (SPM) may contribute to persistent inflammation in CRS.	Vickery et al. (36)
Tissue eCRSwNP vs neCRSwNP vs CRSsNP vs Control	LC-LTQ-Orbitrap-MS	Different subtypes of CRS disease have different metabolomic profiles	Li et al. (33)
Serum eCRSwNP vs neCRSwNP vs Control	UHPLC-ESI-Triple-TOF-MS	Citrulline, linoleic acid, adenosine and 4-guanidinobutyric acid could be potential diagnostic biomarkers of eCRSwNP. And the pathways include arginine and proline metabolism; linoleic acid metabolism.	Xie et al. (35)
Tissue eCRSwNP vs neCRSwNP vs Control	UHPLC-Q-TOF MS	Linoleic acid may be associated with polyp-derived eosinophils, and the mechanism needs further confirmation.	Ma et al. (37)

LC-LTQ-Orbitrap-MS, liquid chromatography/linear ion traps orbitrap mass spectrometry; UHPLC-ESI-Triple-TOF-MS, ultra-high-performance liquid chromatography/electrospray ionization triple time-of-flight mass spectrometry; UHPLC-Q-TOF MS, ultra-high-performance liquid chromatography/quadrupole time-of-flight mass spectrometry.

Recent studies demonstrated that fatty acid metabolism may be associated with CRSwNP. The inflammatory resolution process in patients with CRSwNP is dependent on specialized pro-resolving lipid mediators (SPM) derived from omega-3 and omega-6 polyunsaturated fatty acids (40). SPM could offer new therapeutic opportunities for CRSwNP patients (41). Maresins are a family of essential fatty acid-derived lipid mediators that display protective activities in airway inflammation. Beegun et al. found that in plasma, Maresins’ concentrations were significantly downregulated in the CRSwNP patient group, whereas in nasal secretions, Maresins’ concentrations were significantly upregulated in the CRSwNP patient group, by comparing the lipid profiles of CRSwNP patients and controls (i.e., healthy volunteers, patients with an upper respiratory tract infection) (38). These mediators were associated with quality-of-life scores, which may indicate the potential of Maresins as diagnostic biomarkers of CRSwNP. Additionally, in incubated peripheral blood cells, it was found that Maresins 1 could regulate phagocyte activation, which may indicate pro-resolving therapeutics contribute to limiting inflammation in CRSwNP patients. Miyata et al. found significant dysregulation of fatty acid metabolism in eosinophils in nasal polyps of eCRSwNP patients through a multi-omics study (39). Lipidomics revealed a unique lipid mediator profile in eCRSwNP patients, cysteinyl leukotriene (CysLT) biosynthetic pathway products, leukotriene D4 (LTD4) was much enhanced and LTC4 and E4 were significantly decreased. And eCRSwNP patients with CysLT-related enzymes in proteomics and transcriptomics, gamma-glutamyltransferase 5 and arachidonate 5 lipoxygenase-activating protein were upregulated, while dipeptidase-2 was downregulated. These results reveal that eCRSwNP patients have a unique inflammatory phenotype of dysregulated fatty acid metabolism. Enzymes and metabolites associated with fatty acid metabolism may be potential therapeutic targets. Additionally, by comparing the targeting lipid profiles of CRSwNP, CRSsNP, and control, Vickery et al. found that resolvin D2 was upregulated in both CRSwNP (p=0.00076) and CRSsNP (p=0.030) compared with controls and lipoxin A4 was significantly increased in CRSwNP compared with CRSsNP (p=0.000033) and controls (p=0.044) (36). These results suggested that dysregulated SPM may contribute to persistent inflammation in CRS. Li et al. found elevated levels of unsaturated fatty acid oxidation in eCRSwNP patients, increased uric acid in neCRSwNP patients, and decreased 3-adenosine monophosphate in CRSsNP patients compared to controls (33). They also conducted a follow-up on the CRS patients after surgery, and 9 of the patients had developed refractory CRS. Further study showed an upregulation of glutathione disulfide (GSSG) in these refractory CRS patients. Notably, GSSG could serve as a reliable predictor for refractory CRS (AUC = 0.832). Furthermore, the accumulation of GSSG exacerbates inflammation, which may explain the upregulated GSSG profile in patients with refractory CRS (42). These results indicated that different subtypes of CRS disease have different metabolomic profiles.

To gain more insight into the pathological process of CRSwNP, more and more studies are focusing on eCRSwNP and neCRSwNP. Xie et al. compared the serum metabolomics profiles of eCRSwNP patients, and neCRSwNP patients, and found that citrulline, linoleic acid, adenosine, and 4-guanidinobutyric acid exhibited good accuracy for distinguishing eCRSwNP (AUC > 0.7), and that these significant metabolites related metabolic pathways included arginine and proline metabolism; linoleic acid metabolism; and purine metabolism (35). Especially, linoleic acid levels were negatively correlated with tissue eosinophils percentage. Another study investigating the nasal polyps’ tissues of eCRSwNP by metabolomics reported similar results: linoleic acid levels were negatively correlated with the eosinophil count and percentage in the polyp tissue (37). And the study suggested that linoleic acid contributed most to the differentiation of the eCRSwNP and other groups.

In conclusion, recent studies showed that dysregulated fatty acid metabolism may contribute to the development of nasal polyps in CRSwNP, and Maresins could be potential diagnostic biomarkers and therapeutic targets. SPM could be potential therapeutic targets as well. Additionally, some studies focused on eCRSwNP and found that linoleic acid may be a potential diagnostic biomarker for eCRSwNP.

## 4 Conclusions and perspectives

This review is the first to summarize MS-based proteomics and metabolomics in the field of CRSwNP. Firstly, the review describes the diverse protein and metabolite extraction methods used in studies. Secondly, the review summarizes the most recent mass spectrometry-based CRSwNP proteomics and metabolomics studies from 2019 to 2022. In proteomic studies, potential diagnostic biomarkers like apolipoprotein E, periostin, and Mucin 5AC as potential diagnostic biomarkers for CRSwNP were identified, while other studies reported a range of protein biomarkers such as IgE, cytokines (i.e., IL-4, IL-5, IL-13, IL-25, IL-33), TSLP, p-glycoprotein, C-X-C motif chemokine ligand (CXCL)-12/CXCL-13, IgG and IgA autoantibodies, IgE antibody to S. aureus, enterotoxin, matrix metalloproteinases, and oncostatin M (43). Additionally, bone morphogenetic protein-2 was found to be a potential prognostic biomarker for refractory CRSwNP (44). Pertaining to metabolomics, Maresins, SPM, and linoleic acid were recognized as potential diagnostic or therapeutic biomarkers of CRSwNP or eCRSwNP, and GSSG was a potential prognostic biomarker for refractory CRS.

Multi-omics studies combining proteomics and metabolomics are promising for enhancing the understanding of CRSwNP pathological processes. A study demonstrated the potential of combining these two omics to identify associations between amino acid metabolism, mitochondrial dysfunction, and CRSwNP pathogenesis (16). Furthermore, considering the regional and ethnic specificity of eCRSwNP, stratification and diagnosis at the molecular-level could significantly influence patient care and treatment.

Finally, we propose future directions for MS-based omics in CRSwNP to concentrate on the following facets. The first consideration is sample types. Proteomics has seen substantial advancements and nasal mucus dominated the CRSwNP proteomics samples in recent studies. In contrast, metabolomics samples are mostly tissues and plasma, and in the future, more attention should be paid to extracting intact metabolites from non-invasive obtained samples, such as nasal mucus, for analysis. The second consideration is the subtype of CRS. The EPOS 2020 classifies CRS more clearly, with CRSwNP designated as a primary CRS tissue diffuse type 2 endotype. Central compartment allergic disease (CCAD) and allergic fungal rhinosinusitis (AFRS) also fall under this category. However, there is a paucity of proteomic and metabolomic research on these two subtypes, thus making it a focus area. The third consideration is the small sample cohort. Many studies have the limitation of a small sample cohort. As a non-invasive sample, nasal mucus is more accessible, so future efforts should aim at improving collection methods and the protocols for target extraction of nasal mucus. This allows for the collection and analysis of large cohort samples to draw more universal conclusions. The fourth consideration is the validation of results. After performing untargeted multi-omics analysis, *in vivo* or *in vitro* experiments should be performed for validation. The last one is the implementation of spatially resolved metabolomics techniques. Mass spectrometry imaging techniques are used to discern associations between metabolite distribution and pathological changes within tissues.

## Author contributions

SG: Writing – original draft, Writing – review & editing. MT: Writing – review & editing. YF: Writing – review & editing. XZ: Writing – review & editing.

## References

[1] Fokkens WJ Lund VJ Hopkins C Hellings PW Kern R Reitsma S. European position paper on rhinosinusitis and nasal polyps 2020. Rhinology (2020) 58(Suppl S29):1–464. doi: 10.4193/Rhin20.600 32077450

[2] Sedaghat AR Kuan EC Scadding GK. Epidemiology of chronic rhinosinusitis: prevalence and risk factors. J Allergy Clin Immunol In practice. (2022) 10(6):1395–403. doi: 10.1016/j.jaip.2022.01.016 35092822

[3] Laidlaw TM Mullol J Woessner KM Amin N Mannent LP. Chronic rhinosinusitis with nasal polyps and asthma. J Allergy Clin Immunol In practice. (2021) 9(3):1133–41. doi: 10.1016/j.jaip.2020.09.063 33065369

[4] Eschenbacher W Straesser M Knoeddler A Li RC Borish L. Biologics for the treatment of allergic rhinitis, chronic rhinosinusitis, and nasal polyposis. Immunol Allergy Clinics North America. (2020) 40(4):539–47. doi: 10.1016/j.iac.2020.06.001 PMC7539135 33012318

[5] Tomassen P Vandeplas G Van Zele T Cardell LO Arebro J Olze H. Inflammatory endotypes of chronic rhinosinusitis based on cluster analysis of biomarkers. J Allergy Clin Immunol (2016) 137(5):1449–56.e4. doi: 10.1016/j.jaci.2015.12.1324 26949058

[6] Lou H Meng Y Piao Y Zhang N Bachert C Wang C. Cellular phenotyping of chronic rhinosinusitis with nasal polyps. Rhinology (2016) 54(2):150–9. doi: 10.4193/Rhino15.271 26747641

[7] Wei B Liu F Zhang J Liu Y Du J Liu S. Multivariate analysis of inflammatory endotypes in recurrent nasal polyposis in a Chinese population. Rhinology (2018) 56(3):216–26. doi: 10.4193/Rhin17.240 29785413

[8] Lou H Zhang N Bachert C Zhang L. Highlights of eosinophilic chronic rhinosinusitis with nasal polyps in definition, prognosis, and advancement. Int Forum Allergy Rhinol (2018) 8(11):1218–25. doi: 10.1002/alr.22214 PMC6282610 30296011

[9] Rogawski R Sharon M. Characterizing endogenous protein complexes with biological mass spectrometry. Chem Rev (2021) 122(8):7386–414. doi: 10.1021/acs.chemrev.1c00217 PMC9052418 34406752

[10] Karayel O Virreira Winter S Padmanabhan S Kuras YI Vu DT Tuncali I. Proteome profiling of cerebrospinal fluid reveals biomarker candidates for Parkinson's disease. Cell Rep Med (2022) 3(6):100661. doi: 10.1016/j.xcrm.2022.100661 35732154 PMC9245058

[11] Zhong LP Cheng F Lu XY Duan YX Wang XD. Untargeted saliva metabonomics study of breast cancer based on ultra performance liquid chromatography coupled to mass spectrometry with HILIC and RPLC separations. Talanta (2016) 158:351–60. doi: 10.1016/j.talanta.2016.04.049 27343615

[12] Kao SS Bassiouni A Ramezanpour M Chegeni N Colella AD Chataway TK. Scoping review of chronic rhinosinusitis proteomics. Rhinology (2020) 58(5):418–29. doi: 10.4193/Rhin20.034 32500870

[13] Ninomiya T Noguchi E Haruna T Hasegawa M Yoshida T Yamashita Y. Periostin as a novel biomarker for postoperative recurrence of chronic rhinosinitis with nasal polyps. Sci Rep (2018) 8(1):11450. doi: 10.1038/s41598-018-29612-2 30061580 PMC6065353

[14] Kim YS Han D Kim J Kim DW Kim YM Mo JH. In-depth, proteomic analysis of nasal secretions from patients with chronic rhinosinusitis and nasal polyps. Allergy Asthma Immunol Res (2019) 11(5):691–708. doi: 10.4168/aair.2019.11.5.691 31332980 PMC6658407

[15] Ryu J Thomas SN. Quantitative mass spectrometry-based proteomics for biomarker development in ovarian cancer. Molecules (Basel Switzerland). (2021) 26(9):2674. doi: 10.3390/molecules26092674 PMC8125593 34063568

[16] Yang Y Guo J Yao Y Wang J Yin J Guo Y. Proteomics and metabolomics analysis of nasal lavage fluid in chronic rhinosinusitis with nasal polyps. Int Forum Allergy Rhinol (2023) 1–5. doi: 10.1002/alr.23151 36898695

[17] Farajzadeh Deroee A Oweinah J Naraghi M Hosemann W Athari B Völker U. Regression of polypoid nasal mucosa after systemic corticosteroid therapy: a proteomics study. Am J Rhinol Allergy (2009) 23(5):480–5. doi: 10.2500/ajra.2009.23.3385 19723368

[18] Jung HJ Zhang YL Kim DK Rhee CS Kim DY. The role of NF-κB in chronic rhinosinusitis with nasal polyps. Allergy Asthma Immunol Res (2019) 11(6):806–17. doi: 10.4168/aair.2019.11.6.806 PMC6761067 31552716

[19] Min-man W Hong S Zhi-qiang X Xue-ping F Chang-qi L Dan L. Differential proteomic analysis of nasal polyps, chronic sinusitis, and normal nasal mucosa tissues. Otolaryngol Head Neck Surg (2009) 141(3):364–8. doi: 10.1016/j.otohns.2009.04.022 19716015

[20] Parra-Ferro M Justice JM Lobo BC Munger SD Schlosser RJ Mulligan JK. Utilization of nasal mucus to investigate the pathophysiology of chronic rhinosinusitis. Am J Rhinol Allergy (2022) 36(6):872–83. doi: 10.1177/19458924221111830 PMC12500172 35848564

[21] Kao SS Ramezanpour M Bassiouni A Finnie J Wormald PJ Vreugde S. Barrier disruptive effects of mucus isolated from chronic rhinosinusitis patients. Allergy (2020) 75(1):200–3. doi: 10.1111/all.13964 31254286

[22] Chung YW Cha J Han S Chen Y Gucek M Cho HJ. Apolipoprotein E and periostin are potential biomarkers of nasal mucosal inflammation. A parallel approach of *in vitro* and *in vivo* secretomes. Am J Respir Cell Mol Biol (2020) 62(1):23–34. doi: 10.1165/rcmb.2018-0248OC 31194918 PMC7051477

[23] Simões T Charro N Blonder J Faria D Couto FM Chan KC. Molecular profiling of the human nasal epithelium: A proteomics approach. J Proteomics. (2011) 75(1):56–69. doi: 10.1016/j.jprot.2011.05.012 21621024 PMC7185466

[24] Kim YS Han D Mo JH Kim YM Kim DW Choi HG. Antibiotic-dependent relationships between the nasal microbiome and secreted proteome in nasal polyps. Allergy Asthma Immunol Res (2021) 13(4):589–608. doi: 10.4168/aair.2021.13.4.589 34212546 PMC8255347

[25] Wang LF Lee CH Liang SS Hung CC Wu YR Chien CY. Mucin 5AC is significantly upregulated in exosomes from the nasal lavage fluid and may promote the expression of COX-2, VEGF and MMP-9: an implication in nasal polyp pathogenesis. Rhinology (2021) 59(3):328–36. doi: 10.4193/Rhin20.564 34091656

[26] Zhou M Tan KS Guan WJ Jiang LJ Deng J Gao WX. Proteomics profiling of epithelium-derived exosomes from nasal polyps revealed signaling functions affecting cellular proliferation. Respir Med (2020) 162:105871. doi: 10.1016/j.rmed.2020.105871 32056672

[27] Kao SS Bassiouni A Ramezanpour M Finnie J Chegeni N Colella AD. Proteomic analysis of nasal mucus samples of healthy patients and patients with chronic rhinosinusitis. J Allergy Clin Immunol (2021) 147(1):168–78. doi: 10.1016/j.jaci.2020.06.037 32750382

[28] Soler ZM Schlosser RJ Mulligan JK Smith TL Mace JC Ramakrishan VR. Olfactory cleft mucus proteome in chronic rhinosinusitis: a case-control pilot study. Int Forum Allergy Rhinol (2021) 11(8):1162–76. doi: 10.1002/alr.22743 PMC8670410 33275311

[29] Workman AD Nocera AL Mueller SK Otu HH Libermann TA Bleier BS. Translating transcription: proteomics in chronic rhinosinusitis with nasal polyps reveals significant discordance with messenger RNA expression. Int Forum Allergy Rhinol (2019) 9(7):776–86. doi: 10.1002/alr.22315 30775848

[30] Wang G Zheng H Chen X Zheng J Zhan J Li R. Exploration of predictive biomarkers for postoperative recurrence in chronic rhinosinusitis with nasal polyps based on serum multiple-cytokine profiling. Mediators inflammation. (2022) 2022:1061658. doi: 10.1155/2022/1061658 PMC9534722 36211987

[31] Mueller SK Nocera AL Dillon ST Gu X Wendler O Otu HH. Noninvasive exosomal proteomic biosignatures, including cystatin SN, peroxiredoxin-5, and glycoprotein VI, accurately predict chronic rhinosinusitis with nasal polyps. Int Forum Allergy Rhinol (2019) 9(2):177–86. doi: 10.1002/alr.22226 30485711

[32] Chetwynd AJ Dunn WB Rodriguez-Blanco G. Collection and preparation of clinical samples for metabolomics. Metabolomics: From Fundamentals to Clin Appl (2017) 965:19–44. doi: 10.1007/978-3-319-47656-8_2 28132175

[33] Li JX Wang ZZ Zhai GT Chen CL Zhu KZ Yu Z. Untargeted metabolomic profiling identifies disease-specific and outcome-related signatures in chronic rhinosinusitis. J Allergy Clin Immunol (2022) 150(3):727–35.e6. doi: 10.1016/j.jaci.2022.04.006 35460727

[34] Want EJ Masson P Michopoulos F Wilson ID Theodoridis G Plumb RS. Global metabolic profiling of animal and human tissues *via* UPLC-MS. Nat Protoc (2013) 8(1):17–32. doi: 10.1038/nprot.2012.135 23222455

[35] Xie S Zhang H Liu Y Gao K Zhang J Fan R. The role of serum metabolomics in distinguishing chronic rhinosinusitis with nasal polyp phenotypes. Front Mol Biosci (2020) 7:593976. doi: 10.3389/fmolb.2020.593976 33511154 PMC7835901

[36] Vickery TW Armstrong M Kofonow JM Robertson CE Kroehl ME Reisdorph NA. Altered tissue specialized pro-resolving mediators in chronic rhinosinusitis. Prostaglandins leukotrienes essential Fatty Acids (2021) 164:102218. doi: 10.1016/j.plefa.2020.102218 33338738 PMC7855833

[37] Ma Y Wei Y Liu X Dang H Zou H Tian P. Metabolomics analysis of metabolic patterns in chronic rhinosinusitis with nasal polyps. Allergy (2022) 77(2):653–6. doi: 10.1111/all.15179 34783368

[38] Beegun I Koenis DS Alusi G Dalli J. Dysregulated maresin concentrations in plasma and nasal secretions from patients with chronic rhinosinusitis. Front Immunol (2021) 12:733019. doi: 10.3389/fimmu.2021.733019 34531873 PMC8438229

[39] Miyata J Fukunaga K Kawashima Y Watanabe T Saitoh A Hirosaki T. Dysregulated fatty acid metabolism in nasal polyp-derived eosinophils from patients with chronic rhinosinusitis. Allergy (2019) 74(6):1113–24. doi: 10.1111/all.13726 30667533

[40] Serhan CN. Pro-resolving lipid mediators are leads for resolution physiology. Nature (2014) 510(7503):92–101. doi: 10.1038/nature13479 24899309 PMC4263681

[41] Bäck M Yurdagul A Jr. Tabas I Öörni K Kovanen PT. Inflammation and its resolution in atherosclerosis: mediators and therapeutic opportunities. Nat Rev Cardiol (2019) 16(7):389–406. doi: 10.1038/s41569-019-0169-2 30846875 PMC6727648

[42] Shen D Dalton TP Nebert DW Shertzer HG. Glutathione redox state regulates mitochondrial reactive oxygen production. J Biol Chem (2005) 280(27):25305–12. doi: 10.1074/jbc.M500095200 15883162

[43] Workman AD Kohanski MA Cohen NA. Biomarkers in chronic rhinosinusitis with nasal polyps. Immunol Allergy Clinics North America. (2018) 38(4):679–92. doi: 10.1016/j.iac.2018.06.006 PMC6201304 30342588

[44] Kim JY Lim S Lim HS Kim YS Eun KM Khalmuratova R. Bone morphogenetic protein-2 as a novel biomarker for refractory chronic rhinosinusitis with nasal polyps. J Allergy Clin Immunol (2021) 148(2):461–72.e13. doi: 10.1016/j.jaci.2021.02.027 33667477

